# The Prevalence of Marginally Significant Results in Psychology Over Time

**DOI:** 10.1177/0956797619830326

**Published:** 2019-02-21

**Authors:** Anton Olsson-Collentine, Marcel A. L. M. van Assen, Chris H. J. Hartgerink

**Affiliations:** 1 Department of Methodology and Statistics, Tilburg University; 2 Department of Sociology, Utrecht University

**Keywords:** null-hypothesis significance testing, *p* values, marginal significance, APA, over time, open data, open materials

## Abstract

We examined the percentage of *p* values (.05 < *p* ≤ .10) reported as marginally significant in 44,200 articles, across nine psychology disciplines, published in 70 journals belonging to the American Psychological Association between 1985 and 2016. Using regular expressions, we extracted 42,504 *p* values between .05 and .10. Almost 40% of *p* values in this range were reported as marginally significant, although there were considerable differences between disciplines. The practice is most common in organizational psychology (45.4%) and least common in clinical psychology (30.1%). Contrary to what was reported by previous researchers, our results showed no evidence of an increasing trend in any discipline; in all disciplines, the percentage of *p* values reported as marginally significant was decreasing or constant over time. We recommend against reporting these results as marginally significant because of the low evidential value of *p* values between .05 and .10.

Recent failures to reproduce findings of studies (e.g., as in the “Reproducibility Project: Psychology” by the Open Science Collaboration, 2015) have fanned the debate about the claiming of findings on the basis of their statistical significance. In their article “Redefine Statistical Significance,” Benjamin et al. (2018) argued that the standard for claiming new discoveries, *p* < .05, is too low and a leading cause of nonreproducibility and false-positive results, and they proposed to change the standard to *p* < .005. On the other hand, Lakens et al. (2018) argued that researchers should transparently report and justify their significance level, whether it is .05 or something else.

Following up on the debate on the use of significance levels in psychology, we empirically examined the extent to which studies in psychology claim a finding on the basis of a significance level that is even lower than .05, often called *marginally significant*, that is, .05 < *p* ≤ .10. More specifically, we examined the percentage of *p* values between .05 and .10 that is reported in studies as marginally significant, across journals and disciplines of psychology and over time. On the way, we also reexamined Pritschet, Powell, and Horne’s (2016) claims that marginally significant results have become more prevalent in psychology over time and that results are reported as marginally significant more frequently in social psychology than in developmental psychology. Examining the prevalence of results reported as marginally significant and reexamining the claims of Pritschet et al. is important as it bears on differences in reproducibility across disciplines and trends over time; higher *p* values are generally associated with lower reproducibility and more false positives (Camerer et al., 2016; Ioannidis, 2005; Open Science Collaboration, 2015).

Pritschet et al. (2016) looked at the frequency of articles in which at least one result was reported as marginally significant or as approaching significance in articles from the journals *Cognitive Psychology, Developmental Psychology*, and the *Journal of Personality and Social Psychology*, meant to “represent three major subfields of psychology: cognitive, developmental, and social” (p. 1037), for the years 1970, 1980, 1990, 2000, and 2010. Although Pritschet et al.’s findings may be interpreted as a higher willingness of researchers over time and in social psychology to claim marginal significance in their articles, we should be careful because of the presence of confounding factors. Their outcome variable was the percentage of articles in which at least one result was reported as marginally significant. However, if an article contains more *p* values, the probability increases that the article contains at least one result reported as marginally significant. In devising their outcome measure, Pritschet et al. did not take into account that the number of reported *p* values per journal article has increased over the years or that articles in the *Journal of Personality and Social Psychology*, on average, contain more *p* values than those in (at least) *Developmental Psychology* (Nuijten, Hartgerink, van Assen, Epskamp, & Wicherts, 2016). In further analyses, Pritschet et al. also controlled for the number of experiments in an article, which did not affect their conclusions, but the number of experiments is only a rough and imperfect proxy for the number of *p* values. More generally, any factor affecting the distribution of *p* values and their frequency in the interval .05 to .10, such as the statistical power of research, *p* hacking, or merely the reporting of statistical results, will affect the percentage of articles reporting one or more results as marginally significant. Thus, this outcome provides limited information on researchers’ usage of the concept of marginal significance, both over time and across journals. Factors affecting the distribution of *p* values, however, will not affect the percentage of *p* values between .05 and .10 reported as marginally significant, as this percentage is conditional on the occurrence of such a *p* value.

Whole parts of the scientific literature can be examined using automated methods. Several recent publications have successfully used extracted statistics to examine the scientific literature on the basis of such automated methods (e.g., Lakens, 2015; Nuijten et al., 2016; Vermeulen et al., 2015). One of the most common automated methods is using so-called regular expressions that search through the provided article for predefined strings of text, the results of which are then saved to a data file for analysis. The more complex the data that need to be extracted, the more limited this method becomes. Fortunately, when *p* values are extracted, only three things need to be identified in the text: the *p*, the comparison sign, and the value itself (for an extensive treatment on the limitations of using reported *p* values, see Hartgerink, van Aert, Nuijten, Wicherts, & van Assen, 2016; Jager & Leek, 2014, and discussions in the first issue of Volume 15 of *Biostatistics*). The advantage of automated methods when examining the scientific literature is that they permit collecting large samples of data. For example, Nuijten et al. (2016), using an R package (*statcheck*) that extracts only complete American Psychological Association (APA)-formatted test results (*t, F*, etc.), collected 258,105 *p* values from 30,717 articles published between 1985 and 2013.

Using automated extraction of *p* values, we examined the prevalence of *p* values between .05 and .10 reported as marginally significant in psychology. We first partially replicated and extended Pritschet et al.’s (2016) findings by examining the prevalence of marginally significant results in two journals, the *Journal of Personality and Social Psychology* and *Developmental Psychology*. Then, we examined that prevalence between 1985 and 2016 in journals published by the APA, distinguishing nine psychology disciplines: social, developmental, cognitive, clinical, educational, experimental, forensic, health, and organizational.

## Method

All code and data for this project are available at osf.io/28gxz. We provide links to the relevant code files on the Open Science Framework (OSF) below. We ran all analyses using R (Version 3.4.1; R Core Team, 2017).

### Data

We reused downloaded articles from Hartgerink (2016), consisting of 74,489 articles published between 1985 and 2016 in 74 APA journals (80% of currently existing APA journals). We limited ourselves to data from journals belonging to the APA, which characterizes the following nine disciplines of psychology: “basic/experimental psychology,” “clinical psychology,” “developmental psychology,” “educational psychology, school psychology, and training,” “forensic psychology,” “health psychology and medicine,” “industrial/organizational psychology and management,” “neuroscience and cognition,” and “social psychology and social processes.” The APA characterizes journals into one additional category (“core of psychology”). However, this category consists of journals that publish on general or interdisciplinary psychology; hence, we do not consider it a discipline in psychology and excluded entries unique to it from our final data set. Four journals and 996 articles were unique to this category and were thus excluded. For a detailed summary of journals that were and were not included in our sample and their division into topics and disciplines, see “Journals and APA Topics” in the Supplemental Material available online.

As Hartgerink (2016) downloaded only articles in HTML format, the time span for each journal depends on the year that articles became available in HTML format. We converted them into raw text using the python tool *html2text* (osf.io/4yqhj/; pypi.python.org/pypi/html2text). We extracted the following information from each article using regular expressions (osf.io/qaw74/): digital object identifier (DOI; when available), raw text of the *p* values (e.g., “p=.048”), sign of the *p*-value comparison (>, <, or =), the *p* value itself, the 200 characters preceding the reported *p* value, and the 200 characters immediately succeeding the reported *p* value. We collated these 790,206 entries into one data set, with one entry pertaining to results of one *p* value (osf.io/f3mga/). Thus, our analysis and reported results pertain to those 44,200 articles (see also Table 1) that contained at least one *p* value.

**Table 1.** Summary of Data per Discipline

Discipline	Number of journals	Number of articles with *p* values	Number of *p* values	Number of *p* values per article	Number of *p* values in the range .05 < *p* ≤ .10	Number of *p* values in the range .05 < *p* ≤ .10 per article	Marginal significance (%)^a^	Marginal significance in article (%)^b^
All APA journals	70	44,200	777,596	17.59	42,504	0.96	39.60 [39.13, 40.06]	19.63 [19.26, 20.00]
Clinical	30	15,216	195,999	12.88	10,173	0.67	30.08 [29.19, 30.97]	12.22 [11.70, 12.74]
Cognitive	10	7,882	161,614	20.50	9,343	1.19	39.49 [38.5, 40.49]	23.59 [22.65, 24.52]
Developmental	8	5,624	84,946	15.10	4,181	0.74	37.72 [36.25, 39.19	17.37 [16.38, 18.36]
Educational	10	9,808	143,178	14.60	6,691	0.68	34.69 [33.55, 35.83]	14.07 [13.38, 14.76]
Experimental	19	15,387	334,743	21.75	18,907	1.23	40.65 [39.95, 41.35]	24.55 [23.87, 25.23]
Forensic	4	2,075	26,527	12.78	1,271	0.61	33.91 [31.31, 36.51]	11.42 [10.05, 12.79]
Health	25	11,054	138,266	12.51	6,802	0.62	31.58 [30.47, 32.68]	11.51 [10.91, 12.10]
Organizational	13	10,514	210,732	20.04	12,255	1.17	45.38 [44.5, 46.26]	24.20 [23.38, 25.01]
Social	25	13,746	266,015	19.35	15,736	1.14	44.47 [43.69, 45.25]	25.32 [24.6, 26.05]

Note: Values in brackets are 95% confidence intervals. APA = American Psychological Association.

a This column shows the percentage of *p* values greater than .05 but less than or equal to .10 reported as marginally significant. ^b^This column shows the percentage of articles containing *p* values with at least one *p* value greater than .05 but less than or equal to .10 reported as marginally significant.

Using the same data set as Hartgerink (2016), we also extracted information on the degrees of freedom across disciplines for a supplementary analysis of statistical power in psychology articles. To do so, we used the R package *statcheck* (Version 1.2.2; Epskamp & Nuijten, 2016), extracting 521,475 APA-formatted statistical results. As this analysis required strong assumptions (i.e., assuming similar true effect-size distributions and designs across disciplines and over time) and was relevant only for the percentage of articles containing at least one result reported as marginally significant, we report further on these data only in the Supplemental Material.

### Data preparation

**Fig. 1.**
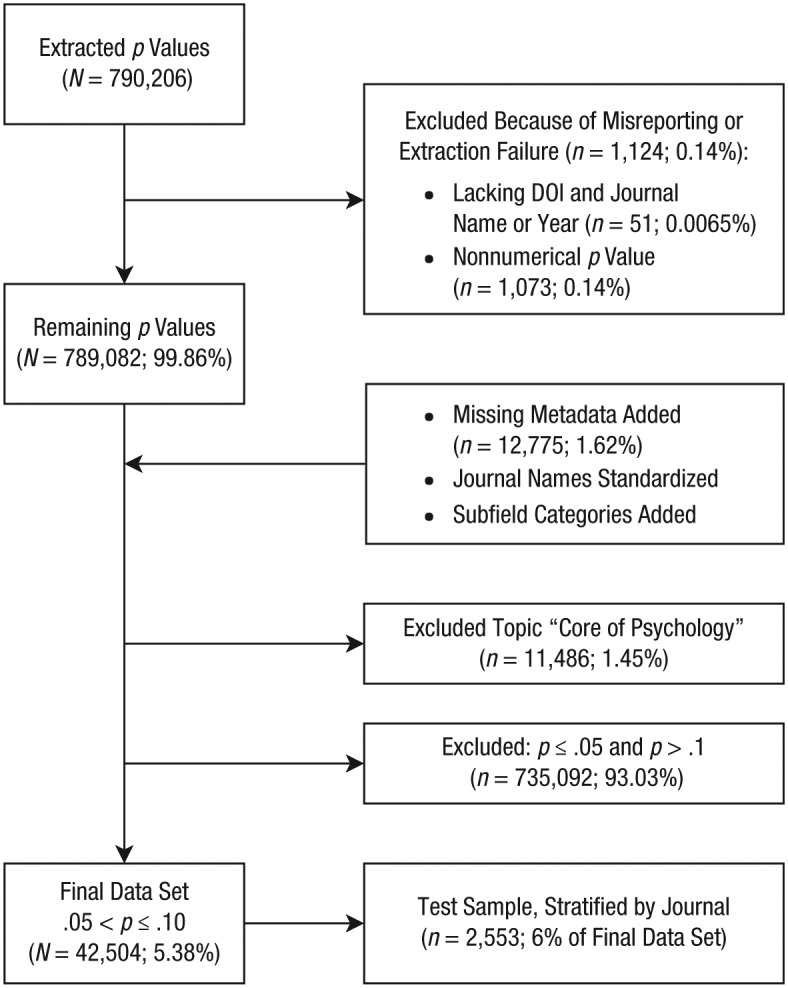
Flowchart illustrating the process generating the test sample and the final data set. DOI = digital object identifier.

We excluded a small number of entries from the extracted data because of misreporting or extraction failure (for a flowchart, see Fig. 1). We removed entries lacking a DOI (and journal name and year; *n* = 51, 0.01% of total) and all entries in which the *p* values were not numerical (e.g., equal to “.”; *n* = 1,073, 0.14% of total; osf.io/gzyt9/); *p* values that were misreported as too high (e.g.,p=1.2 instead of p=.12) were excluded as well as all other *p* values above .10 at a later stage (see below). Note that a few misreported *p* values remain in the data set, for example, those misreported as p=.099 instead of p=.99.

Subsequently, we added discipline information to each entry. Before adding this information, we used the R package *rcrossref* (Version 0.6.0; Chamberlain, Boettiger, Hart, & Ram, 2016) to retrieve missing metadata (years and journal name) for all entries lacking such data (*n* = 1.62% of the total; osf.io/gzyt9/). We also standardized journal names for all entries, with older journal names updated to their current APA names (as of 2017; see “Journals and APA Topics”; osf.io/gzyt9/). We then added dummies for each discipline to all entries (osf.io/gzyt9/).

Finally, we excluded the topic core of psychology and all *p* values outside the range of .05 to .10, and we created a test sample. We excluded 11,486 (1.45% of total) entries unique to the topic core of psychology (osf.io/gzyt9/). Limiting the data set to *p* values greater than .05 but less than or equal to .10 resulted in a final sample of 42,504 (5.38% of the total) *p* values (osf.io/gzyt9/). From the final data set, we drew a stratified random sample of 6% per journal for the testing code used for data analysis (osf.io/y953k/). For our analyses reported below, we used the full final data set, including the test sample data.

Table 1 summarizes the data per discipline. As per the APA’s categorization, a journal may belong to multiple disciplines (see also “Journals and APA Topics”). A *p* value in an article is part of the *p*-value count for each discipline that it belongs to. To determine whether a result was reported as marginally significant, we searched the 200 characters preceding and the 200 characters succeeding a given *p* value for the expressions “margin*” and “approach*” (following Pritschet et al., 2016), using regular expressions, and considered the *p* value to be reported as marginally significant if either of those expressions was found. We also reported the percentage of articles containing *p* values per discipline in which at least one *p* value between .05 and .10 was reported as marginally significant (last column).

Table 2 shows a comparison of our data with the data provided by Pritschet et al. (2016, available at osf.io/92xqk) with respect to the two APA journals (*Developmental Psychology* and *Journal of Personality and Social Psychology*) that their article and ours have in common. Pritschet et al. concerned themselves with whether an article contained a marginally significant result, which is not necessarily associated with a *p* value between .05 and .10 (92.6% of their marginal *p* values fell between .05 and .10), and consequently, each row in their data set represents a different article. Their data do not include the total number of *p* values or the number of *p* values between .05 and .10 in their sample.

**Table 2.** Comparison Between the Data of Pritschet, Powell, and Horne (2016) and the Data of the Current Article With Respect to the *Journal of Personality and Social Psychology* and *Developmental Psychology*

Article and journal	Time span	Number of articles	Number of *p* values	Number of *p* values per article	Number of *p* values in the range .05 < *p* ≤ .10	Number of *p* values in the range .05 < *p* ≤ .10 per article	Marginal significance (%)^a^	Marginal significance in article (%)^b^
Current article								
*Journal of Personality and Social Psychology*	1985–2016	4,073	114,872	28.20	8,001	1.96	49.88 [48.79, 50.98]	41.84 [40.32, 43.35]
*Developmental Psychology*	1985–2016	2,806	49,201	17.53	2,541	0.91	39.71 [37.81, 41.61]	21.74 [20.21, 23.27]
Pritschet et al. (2016)								
*Journal of Personality and Social Psychology*	1970–2010	873						39.52 [36.28, 42.76]
*Developmental Psychology*	1970–2010	564						24.29 [20.75, 27.83]

Note: Cells for numbers that could not be calculated using the data set provided by Pritschet et al. (2016) have been left blank (see osf.io/92xqk). Values in brackets are 95% confidence intervals.

a This column shows the percentage of *p* values greater than .05 but less than or equal to .10 reported as marginally significant. ^b^This column shows the percentage of articles containing *p* values with at least one *p* value greater than .05 but less than or equal to .10 reported as marginally significant.

### Analyses

Because we used a nonrandom sample (only APA articles available in HTML format at the time of download) and dependent samples (many *p* values are included in multiple disciplines), we focused on descriptive statistics and conducted no inferential statistical tests. As per journal standards, we nonetheless report 95% confidence intervals in tables and figures for estimates (osf.io/xyh8n/) but caution against interpreting these inferentially.

We describe trends in percentages of marginally significant results across years and disciplines and for the *Journal of Personality and Social Psychology* and *Developmental Psychology* separately (osf.io/wa62v/). To aid interpretation, we estimated and report slopes of 24 simple linear regressions using least squares: two for each of the nine disciplines, two across all disciplines, and two each for the *Journal of Personality and Social Psychology* and *Developmental Psychology*. The outcome variable in these regressions is the percentage of *p* values (.05 < *p* ≤ .10) reported as marginally significant per year in each category or the percentage of articles containing *p* values with at least one result (.05 < *p* ≤ .10) reported as marginally significant. The independent variable is the year (range = 1985–2016) of publication of the articles from which the *p* values were extracted. In addition, we report averages across the years for each category (osf.io/79t2p/).

## Results

We present our results in two steps. First, we present results for the *Journal of Personality and Social Psychology* and *Developmental Psychology*. Here, we also considered the average number of *p* values between .05 and .10 reported per article and year. Second, we present the results for all included APA journals taken together and for the nine psychology disciplines previously described (see Table 1).

### *Journal of Personality and Social Psychology* and *Developmental Psychology*

**Fig. 2.**
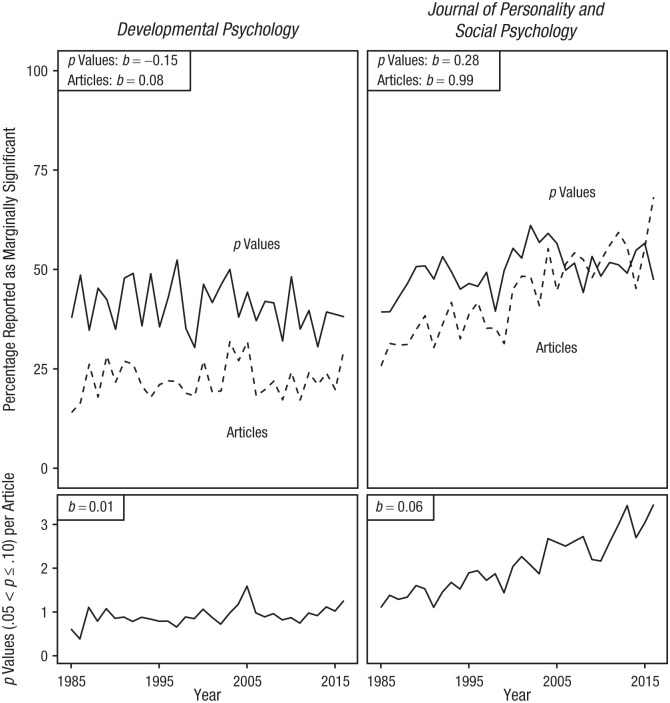
Results for *Developmental Psychology* and the *Journal of Personality and Social Psychology*. The top row shows the percentage of *p* values (.05 < *p* ≤ .10) reported as marginally significant and percentage of articles with *p* values containing at least one such marginally significant *p* value between 1985 and 2016. The bottom row shows the number of reported *p* values (.05 < *p* ≤ .10) per article, given that the articles contained at least one *p* value. Slope coefficients (*b*s) are reported from simple linear regressions.

Our analyses confirmed that the percentage of articles with at least one result reported as marginally significant was higher in the *Journal of Personality and Social Psychology* than in *Developmental Psychology*; whereas Pritschet et al. (2016) found percentages of 39.52 (  *Journal of Personality and Social Psychology*) and 24.29 (*Developmental Psychology*), we found percentages of 41.84 and 21.74, respectively (see Table 2, last column). The differences (albeit small) between their and our results are explained by the fact that we incorporated other articles and by differences in the selection and calculation of results (marginally significant results by Pritschet et al. and *p* values in the .05–.10 range in combination with a window of ±200 words). Following Pritschet et al., we observed an increase in the reporting of marginally significant results at the level of articles for *Developmental Psychology* and the *Journal of Personality and Social Psychology*, although the increase for *Developmental Psychology* was very small (estimated increase of approximately 2.5% over 30 years; see Fig. 2). For the *Journal of Personality and Social Psychology*, this trend was brought about by an increase in both the average number of *p* values between .05 and .10 per article and the percentage of *p* values between .05 and .10 reported as marginally significant (see Fig. 2). For *Developmental Psychology*, the percentage of *p* values reported as marginally significant decreased over time, but this decrease was offset by a larger increase in the number of *p* values between .05 and .10 over time. The latter results demonstrate the importance of distinguishing results at the level of articles from those at the level of *p* values.

### Psychology and its disciplines

Reporting *p* values between .05 and .10 as marginally significant was common practice in all psychology disciplines. Table 2 shows that, on average, almost 40% of *p* values (.05 < *p* ≤ .10) in the 70 examined APA journals were reported as marginally significant between 1985 and 2016. The practice was most common in organizational psychology (45.38%), social psychology (44.47%), and experimental psychology (40.65%). The fewest *p* values between .05 and .10 were reported as marginally significant in clinical psychology (30.08%), health psychology (31.58%), and forensic psychology (33.91%). The disciplines of educational psychology (34.69%), developmental psychology (37.72%), and cognitive psychology (39.49%) fell between these two groups. That higher percentages were consistently found for the outcome variable at the level of *p* values (see Table 2, penultimate column) than at the level of articles (last column) is explained by the many articles that contain *p* values but without values in the range .05 to .10. Of the total 44,200 articles with *p* values, only 25,800 contained *p* values between .05 and .10, which thus inflates the denominator of the percentage of articles containing at least one marginally significant result.

**Fig. 3.**
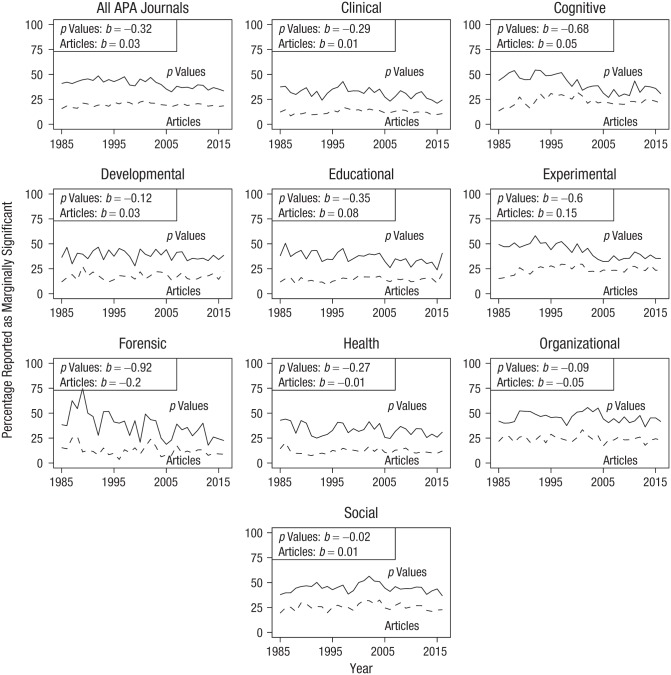
Percentage of *p* values (.05 < *p* ≤ .10) reported as marginally significant (solid lines) and percentage of articles containing at least one such *p* value (dashed lines) between 1985 and 2016 in different psychology disciplines with data extracted from American Psychological Association (APA) journals. Slope coefficients (*b*s) are reported from simple linear regressions for each discipline and overall.

We examined the overall trend in the reporting of marginally significant results and the trends in each discipline (see Fig. 3). Across all journals, the percentage of *p* values reported as marginally significant decreased (*b* = −0.32) in the period from 1985 to 2016. For no discipline was there evidence of an increasing trend. On the basis of the linear trend (*b*), the largest decreases were in forensic psychology (*b* = −0.92), cognitive psychology (*b* = −0.68), and experimental psychology (*b* = −0.6). Three disciplines were mostly stable over the years: social psychology (*b* = −0.02), organizational psychology (*b* = −0.09), and developmental psychology (*b* = −0.12). The change over time for the three remaining disciplines fell between these two groups. These were health psychology (*b* = −0.27), clinical psychology (*b* = −0.29), and educational psychology (*b* = −0.35). Note that the plots also indicate a trend for more *p* values reported in the literature.

The percentage of articles containing *p* values with at least one *p* value between .05 and .10 reported as marginally significant increased when averaged across all APA journals and for all disciplines individually, except for forensic psychology, health psychology, and organizational psychology (see Fig. 2). As demonstrated in the previous section, these trends are not straightforward to interpret, as they are also affected by trends in the frequency of *p* values between .05 and .10 per article. Consecutively, this frequency of *p* values is affected by trends in the reporting of *p* values and trends in the statistical power of psychological research over time, although there is, at most, a small increase in power over time in our data (see the Supplemental Material). Note that possible trends in *p*-value reporting and power do not affect the percentage of *p* values reported as marginally significant, as that percentage is conditional on the *p* value being between .05 and .10.

## Discussion

Following up on the debate about the use of significance levels in psychology, we empirically examined the extent to which researchers have claimed a finding to be marginally significant on the basis of a *p* value between .05 and .10 in psychology and its disciplines between 1985 and 2016. Examining the prevalence of results reported as marginally significant is important, as it bears on differences in reproducibility across disciplines and trends over time; higher *p* values are generally associated with lower reproducibility and more false positives. Following Pritschet et al. (2016), we examined trends in the percentage of articles with *p* values reported as marginally significant and showed that these are affected by differences across disciplines in the number of *p* values between .05 and .10 and the development over time of this number. We also examined the prevalence of *p* values between .05 and .10 reported as marginally significant across time in nine psychology disciplines, which is not affected by factors influencing the distribution of *p* values.

That *p* values between .05 and .10 are interpreted as marginally significant appears common in psychology. Across the nine disciplines we examined, almost 40% of such values were reported as marginally significant in the period from 1985 to 2016, although the prevalence differed by discipline. We found higher percentages of *p* values between .05 and .10 reported as marginally significant in social psychology than in developmental and cognitive psychology, corroborating the findings by Pritschet et al. (2016), but differences were small (up to 7%). Overall, marginally significant *p* values were the most prevalent in organizational psychology and the least prevalent in clinical psychology.

A few disciplines had a stable trend, but most described a downward trend in the percentage of *p* values between .05 and .10 reported as marginally significant between 1985 and 2016. Controlling for the increasing numbers of *p* values across the years, we found that the positive trends reported by Pritschet et al. (2016) for cognitive psychology, developmental psychology, and social psychology thus disappeared. On the other hand, the *Journal of Personality and Social Psychology*, which Pritschet et al. used to represent social psychology, still showed a positive trend. This illustrates the problem with using a single journal to represent entire psychology disciplines. The downward trend in psychology overall may reflect increasing awareness among researchers that *p* values in the range of .05 to .10 represent weak evidence against the null or a tendency to also report *p* values that do not correspond to tests of the main hypotheses and are not interpreted in the main text. It may also be that percentages are decreasing because of increasingly stringent competition to publish and less leniency among editors for marginally significant results (as previously suggested by Lakens, 2015). Regardless of the reason, what matters is that results with such *p* values do not end up in the file drawer and are not “transformed” into significant results (Simmons, Nelson, & Simonsohn, 2011) but are reported in the literature.

We demonstrated that it is not straightforward to examine and interpret trends in the percentage of articles that report at least one *p* value between .05 and .10 as marginally significant because they are affected by factors influencing the *p*-value distribution of results reported in articles. One can attempt to model the *p*-value distribution and factors influencing it. However, as so many factors affect the *p*-value distribution and these models are based on strong assumptions, we believe it is impossible to draw strong conclusions on the mechanisms causing differences or trends in *p*-value distributions (Hartgerink et al., 2016). We therefore recommend examining the percentage of *p* values between .05 and .10 that is reported as marginally significant, as it is not affected by these factors.

Our results are qualified by three issues. First, because *p* values of .05 tend to be reported as significant (Nuijten et al., 2016), we excluded these results, regardless of whether the sign was >, <, or =. However, a portion of *p* values reported as “*p* > .05” will also be below or equal to .10. It seems possible that researchers who report a *p* value between .05 and .10 as “*p* > .05” would also be less likely to report this result as marginally significant and label it nonsignificant instead. If this is the case, our results may be slightly biased in favor of higher estimates. On the other hand, our second limitation leads to bias in the opposite direction. Matthew Hankins (2013) compiled a list of 508 ways that researchers have described results as marginally significant. Of these, only 77 include the expressions “margin*” or “approach*,” our indicators of marginal significance. Although there is no telling how common the different expressions on Hankins’s list are, their existence nonetheless indicates that our estimates of the prevalence of marginally significant results in psychology are likely to be underestimates because of the varied terminology available to label results that are close to significance. Third, and relatedly, our results on marginal significance are limited by our data-collection procedure; strictly speaking, our conclusions apply to the use of “margin*” and “approach*” in the window of ±200 characters of a *p* value between .05 and .10. To conclude, we cannot blindly generalize our conclusions to the overall use of marginal significance in the psychological literature.

In the end, the degree to which results reported as marginally significant are problematic depends on research design. Questionable research practices inflate the risk of false-positive results (John, Loewenstein, & Prelec, 2012). One of a multitude of such practices is the post hoc decision to change what decision rule one uses or how strictly it is applied (Wicherts et al., 2016). Because most researchers are likely to use an implicitly predefined alpha level, later reporting results as marginally significant is an example of an implicit change in the decision rule. The severity of this practice depends on the extent to which the decision rule has been altered. Nevertheless, because *p* values between .05 and .10 are known to have low evidential value (Benjamin et al., 2018; Ioannidis, 2005), we recommend against reporting these results as being marginally significant.

## Supplemental Material

JournalsandAPATopics – Supplemental material for The Prevalence of Marginally Significant Results in Psychology Over Time Click here for additional data file. Supplemental material, JournalsandAPATopics for The Prevalence of Marginally Significant Results in Psychology Over Time by Anton Olsson-Collentine, Marcel A. L. M. van Assen and Chris H. J. Hartgerink in Psychological Science

## Supplemental Material

OlssonCollentineOpenPracticesDisclosure – Supplemental material for The Prevalence of Marginally Significant Results in Psychology Over Time Click here for additional data file. Supplemental material, OlssonCollentineOpenPracticesDisclosure for The Prevalence of Marginally Significant Results in Psychology Over Time by Anton Olsson-Collentine, Marcel A. L. M. van Assen and Chris H. J. Hartgerink in Psychological Science

## Supplemental Material

SOM – Supplemental material for The Prevalence of Marginally Significant Results in Psychology Over Time Click here for additional data file. Supplemental material, SOM for The Prevalence of Marginally Significant Results in Psychology Over Time by Anton Olsson-Collentine, Marcel A. L. M. van Assen and Chris H. J. Hartgerink in Psychological Science
