# Supplementary material for: The Prevalence of Marginally Significant Results in Psychology Over Time
Source: Psychol Sci. 2019 Feb 21;30(4):576–86. doi: 10.1177/0956797619830326 (PMC6472145; doi:10.1177/0956797619830326)
Supplement: JournalsandAPATopics – Supplemental material for The Prevalence of Marginally Significant Results in Psychology Over Time [file JournalsandAPATopics.pdf]

Journals and APA Topics

"Basic / Experimental Psychology" (experimental psychology) - 19 journals

- Behavioral Neuroscience
- Canadian Journal Of Experimental Psychology
- Decision
- Dreaming
- Emotion
- Experimental And Clinical Psychopharmacology
- Journal Of Comparative Psychology
- Journal Of Experimental Psychology: Applied
- Journal Of Experimental Psychology: General
- Journal Of Experimental Psychology: Human Perception And Performance
- Journal Of Experimental Psychology: Learning, Memory, And Cognition
- Journal Of Neuroscience, Psychology, And Economics
- Motivation Science
- Neuropsychology
- Psychological Methods
- Psychology Of Aesthetics, Creativity, And The Arts

- Psychology Of Consciousness: Theory, Research, And Practice
- Psychology Of Popular Media Culture
- Psychomusicology: Music, Mind, And Brain

"Clinical Psychology" (clinical psychology) - 30 journals

- American Journal Of Orthopsychiatry
- Clinical Practice In Pediatric Psychology
- Couple And Family Psychology: Research And Practice
- Dreaming
- Experimental And Clinical Psychopharmacology
- Health Psychology
- International Journal Of Play Therapy
- International Journal Of Stress Management
- International Perspectives In Psychology: Research, Practice, Consultation
- Journal Of Abnormal Psychology
- Journal Of Consulting And Clinical Psychology
- Journal Of Counseling Psychology
- Journal Of Latina/O Psychology
- Journal Of Psychotherapy Integration

- Journal Of Rural Mental Health
- Journal Of Threat Assessment And Management
- Neuropsychology
- Personality Disorders: Theory, Research, And Treatment
- Psychiatric Rehabilitation Journal
- Psychoanalytic Psychology
- Psychological Assessment
- Psychological Services
- Psychological Trauma: Theory, Research, Practice, And Policy
- Psychology Of Addictive Behaviors
- Psychology Of Sexual Orientation And Gender Diversity
- Psychotherapy
- Rehabilitation Psychology
- Spirituality In Clinical Practice
- Sport, Exercise, And Performance Psychology
- Training And Education In Professional Psychology

"Core of Psychology" (NA, journals unique to this topic (4) excluded from the final dataset) - 9 journals

- American Psychologist
- Canadian Psychology
- History Of Psychology
- International Perspectives In Psychology: Research, Practice, Consultation
- Journal Of Theoretical And Philosophical Psychology
- Psychological Bulletin
- Psychological Methods
- Psychological Review
- Review Of General Psychology

"Developmental Psychology" (developmental psychology) - 8 journals

- Canadian Journal Of Behavioural Science
- Clinical Practice In Pediatric Psychology
- Cultural Diversity & Ethnic Minority Psychology
- Developmental Psychology
- International Journal Of Play Therapy
- Journal Of Family Psychology
- Psychoanalytic Psychology
- Psychology Of Men & Masculinity

"Educational Psychology, School Psychology & Training" (educational psychology) - 10 journals

- Canadian Journal Of Behavioural Science
- Developmental Psychology
- Journal Of Counseling Psychology
- Journal Of Diversity In Higher Education
- Journal Of Educational Psychology
- Neuropsychology
- Psychological Assessment
- Rehabilitation Psychology
- School Psychology Quarterly
- Training And Education In Professional Psychology

"Forensic Psychology" (forensic psychology) - 4 journals

- Journal Of Threat Assessment And Management
- Law And Human Behavior
- Psychological Assessment
- Psychology, Public Policy, And Law

"Health Psychology & Medicine" (health psychology) - 25 journals

- Clinical Practice In Pediatric Psychology
- Cultural Diversity & Ethnic Minority Psychology
- Experimental And Clinical Psychopharmacology
- Health Psychology
- International Journal Of Stress Management
- Journal Of Counseling Psychology
- Journal Of Latina/O Psychology
- Journal Of Occupational Health Psychology
- Journal Of Rural Mental Health
- Military Psychology
- Neuropsychology
- Personality Disorders: Theory, Research, And Treatment
- Psychiatric Rehabilitation Journal
- Psychological Assessment
- Psychological Services
- Psychological Trauma: Theory, Research, Practice, And Policy
- Psychology Of Addictive Behaviors
- Psychology Of Men & Masculinity

- Psychology Of Religion And Spirituality
- Psychology Of Sexual Orientation And Gender Diversity
- Psychology Of Violence
- Rehabilitation Psychology
- Spirituality In Clinical Practice
- Sport, Exercise, And Performance Psychology
- Stigma And Health

"Industrial/Organizational Psychology & Management" (organizational psychology) - 13 journals

- Consulting Psychology Journal: Practice And Research
- Group Dynamics: Theory, Research, And Practice
- International Journal Of Stress Management
- Journal Of Applied Psychology
- Journal Of Experimental Psychology: Applied
- Journal Of Experimental Psychology: General
- Journal Of Neuroscience, Psychology, And Economics
- Journal Of Occupational Health Psychology
- Journal Of Personality And Social Psychology

- Military Psychology
- Psychological Assessment
- Rehabilitation Psychology
- Review Of General Psychology

"Neuroscience & Cognition" (cognitive psychology) - 10 journals

- Behavioral Neuroscience
- Canadian Journal Of Experimental Psychology
- Decision
- Evolutionary Behavioral Sciences
- Journal Of Experimental Psychology: Learning, Memory, And Cognition
- Journal Of Neuroscience, Psychology, And Economics
- Motivation Science
- Neuropsychology
- Psychology Of Aesthetics, Creativity, And The Arts
- Psychomusicology: Music, Mind, And Brain

"Social Psychology & Social Processes" (social psychology) - 25 journals

- American Journal Of Orthopsychiatry
- Canadian Journal Of Behavioural Science

- Canadian Psychology
- Couple And Family Psychology: Research And Practice
- Cultural Diversity & Ethnic Minority Psychology
- Developmental Psychology
- Emotion
- Evolutionary Behavioral Sciences
- Group Dynamics: Theory, Research, And Practice
- History Of Psychology
- International Perspectives In Psychology: Research, Practice, Consultation
- Journal Of Diversity In Higher Education
- Journal Of Family Psychology
- Journal Of Latina/O Psychology
- Journal Of Personality And Social Psychology
- Journal Of Threat Assessment And Management
- Law And Human Behavior
- Peace And Conflict: Journal Of Peace Psychology
- Psychology Of Aesthetics, Creativity, And The Arts
- Psychology Of Men & Masculinity

- Psychology Of Popular Media Culture
- Psychology Of Religion And Spirituality
- Psychology Of Sexual Orientation And Gender Diversity
- Psychology Of Violence
- Psychology, Public Policy, And Law

Journals of the American Psychological Association not in the sample (NA) - 18 journals

- Archives of Scientific Psychology
- Asian American Journal of Psychology
- Behavior Analysis: Research and Practice
- Behavioral Development Bulletin
- Clinician's Research Digest: Adult Populations
- Clinician's Research Digest: Child and Adolescent Populations
- Contemporary Psychology: APA Review of Books
- Families, Systems, & Health
- The Humanistic Psychologist
- Journal of Experimental Psychology: Animal Learning and Cognition
- Practice Innovations
- Professional Psychology: Research and Practice

- The Psychologist-Manager Journal
- Psychology & Neuroscience
- Psychology and Aging
- Qualitative Psychology
- Scholarship of Teaching and Learning in Psychology
- Translational Issues in Psychological Science
- Traumatology
