# Supplementary material for: The Prevalence of Marginally Significant Results in Psychology Over Time
Source: Psychol Sci. 2019 Feb 21;30(4):576–86. doi: 10.1177/0956797619830326 (PMC6472145; doi:10.1177/0956797619830326)
Supplement: SOM – Supplemental material for The Prevalence of Marginally Significant Results in Psychology Over Time [file SOM.pdf]

Supplementary materials: Degrees of freedom over time and across disciplines as a proxy of statistical power

We explore power over time in psychology overall and across the nine different disciplines examined in the main manuscript. To do so we use reported degrees of freedom as a proxy for power. As reported in the main manuscript we used ‘statcheck’ (Epskamp & Nuijten, 2016) to extract APA-formatted statistical results from the dataset of Hartgerink (2016), extracting 521,475 results consisting of *Z*-scores, *F*, *t*, *r* and Chi-square statistics.

We removed a small number of entries lacking DOI (n = 26, 0.005% of total, see Figure S1), and all *Z*-scores and Chi-square statistics (n = 71,474, 13.76% of total). We excluded these entries as they do not provide sufficient information on sample size. Next, we added meta-data, following the same procedure described in the data preparation section of the main manuscript. We also excluded entries unique to the topic ‘Core of psychology’ (n = 3,382, 0.65% of total), which left us with a final dataset consisting of 446,320 entries, where each entry corresponded to an *r*, *t* or *F* statistic.

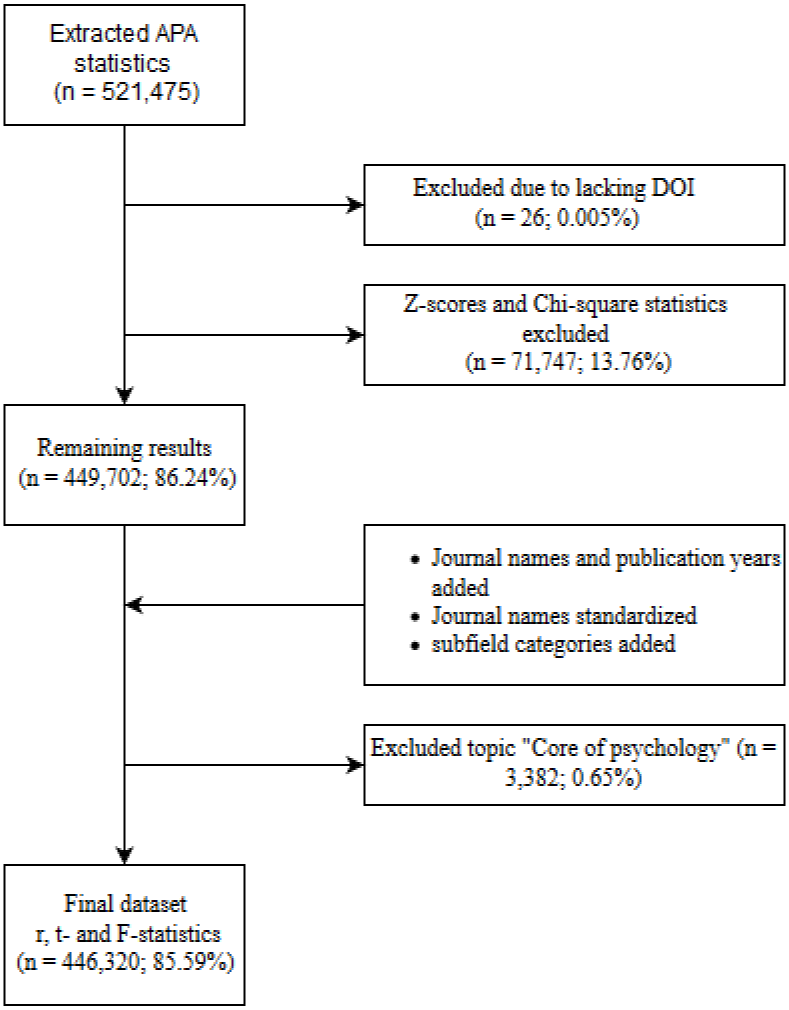

Figure S1. Flowchart illustrating the process generating the final degrees of freedom dataset.

For our analysis we aggregated the extracted statistics by year and calculated the median degrees of freedom for each year and discipline (Figure S2). In the case of *F*-statistics we used the denominator degrees of freedom. We also report ten simple linear regression using least squares, one for each discipline and overall, with the median degrees of freedom as the outcome variable and year (1985 - 2016) as the independent

variable. For plotting, but not for the linear regressions, the median degrees of freedom were averaged over two years for each of the disciplines, due to their large variation from year to year.

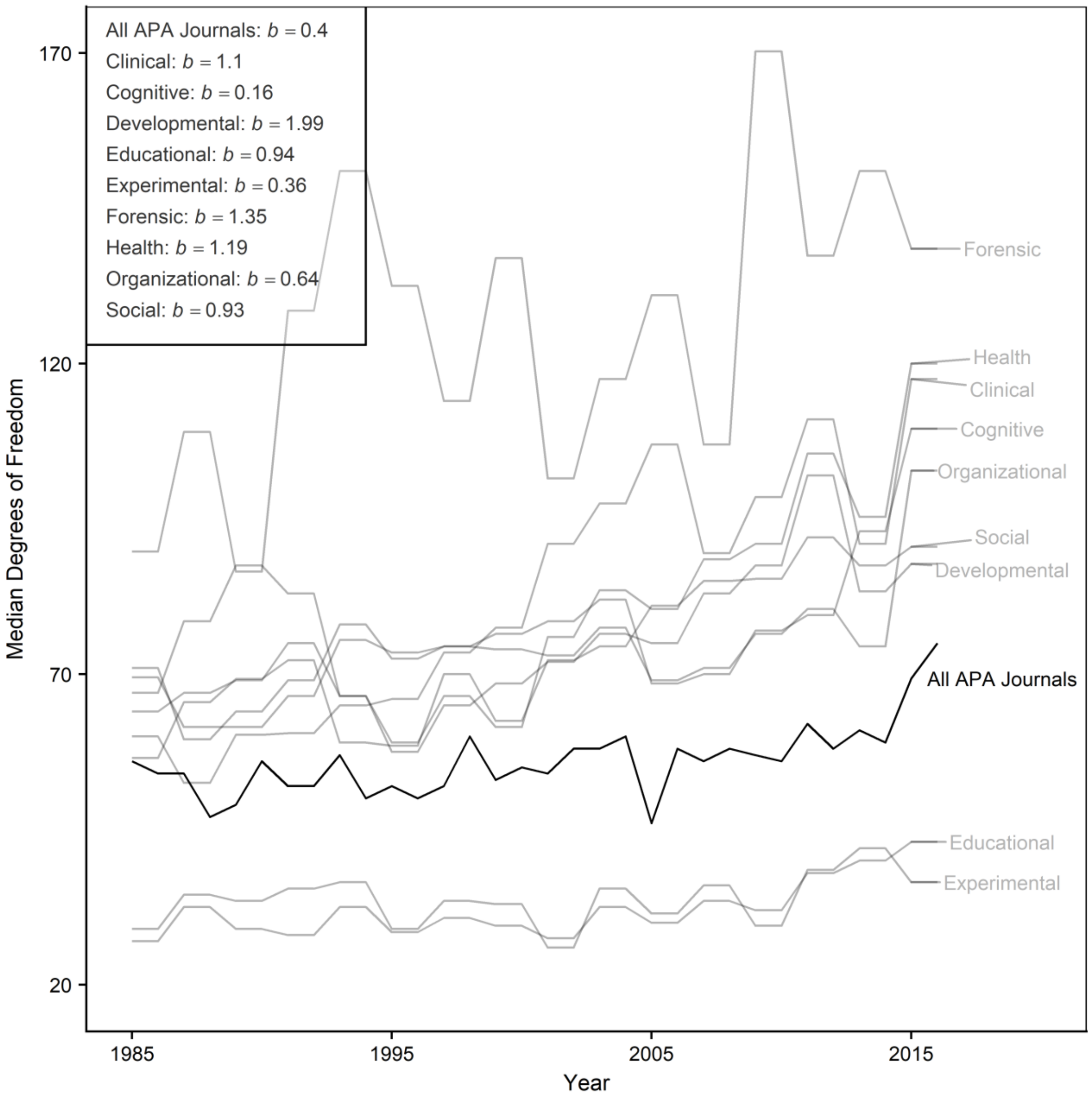

Figure S2. Median degrees of freedom across disciplines over time. Values for disciplines, but not overall, are averages over two years.

As can be seen in Figure S2, overall there is a small increase in the median number of degrees of freedom over time (b = 0.4, corresponding to an increase in df equal to 12, in 30 years), and the degrees of freedom increased in all disciplines over time, the most in Developmental (b = 1.99) and the least in Cognitive (b = 0.16). Using degrees of freedom as a proxy for power, there thus appears to be a weak tendency for higher statistical power over time in psychology, assuming similar true effect size distributions, designs, etc. over time and disciplines. Given these rather strong assumptions, this increase in power should lead to more p-values below .05. Interestingly, the effect of statistical power on the probability of a marginal p-value (i.e., a p-value between .05 and .10) is curvilinear; assuming a normal distribution, for power up to about .42 the probability of a marginal p-value is increasing (with a maximum probability equal to .144), and decreasing for power larger than .42. As the effect of statistical power is not uniformly positive, and statistical power in psychology research is considered to be low on average (approximately 35%; Bakker, Nuijten, and Wicherts, 2012; Stanley, Carter, and Doucouliagos, 2018), we cannot straightforwardly use the association between power and p-values in the marginal range to draw conclusions on its effect on the prevalence of ‘marginally significant’ results at the article level. In addition, other factors which influence p-value distributions, such as p-hacking and publication bias also make it difficult, if not impossible, to draw strong conclusions about the mechanisms that affect p-value distributions (see also Hartgerink et al., 2016). As stated in the main manuscript, power has no effect on our other outcome variable, the percentage of p-values between .05 and .1 reported as marginally significant.

References

Bakker, M., van Dijk, A., & Wicherts, J. M. (2012). The rules of the game called psychological science. *Perspectives on Psychological Science, 7(6)*, 543-554.

Epskamp, S. & Nuijten, M. B. (2016). statcheck: Extract statistics from articles and recompute p values. Retrieved from http://CRAN.R-project.org/package=statcheck. (R package version 1.2.2)

Hartgerink, C. H. J. (2016). 688,112 Statistical Results: Content Mining Psychology Articles for Statistical Test Results. *Data*, *1*(3), 14. doi:10.3390/data1030014

Hartgerink, C. H. J., Aert, R. C. M. van, Nuijten, M. B., Wicherts, J. M., & Assen, M. A. L. M. van. (2016). Distributions of p-values smaller than .05 in psychology: What is going on? *PeerJ*, *4*, e1935. doi:10.7717/peerj.1935.

Stanley, T.D., Carter, E.C., & Doucouliagos, H. (2018). What meta-analyses reveal about the replicability of psychological research. *Psychological Bulletin*.
